# Effects of gastrointestinal parasites on fecal glucocorticoids and behaviour in vervet monkeys (*Chlorocebus pygerythrus*)

**DOI:** 10.1371/journal.pone.0316728

**Published:** 2025-01-30

**Authors:** Pooja Upadhayay, Colin A. Chapman, Gabriela F. Mastromonaco, Valerie A.M. Schoof

**Affiliations:** 1 Department of Biology, Faculty of Graduate Studies, York University, Toronto, Ontario, Canada; 2 Department of Biology, Vancouver Island University, Nanaimo, British Columbia, Canada; 3 Wilson Center, Washington, DC, United States of America; 4 School of Life Sciences, University of KwaZulu-Natal, Pietermaritzburg, South Africa; 5 Shaanxi Key Laboratory for Animal Conservation, Northwest University, Xi’an, China; 6 Wildlife Science, Toronto Zoo, Toronto, Ontario, Canada; 7 Bilingual Biology Program, Department of Multidisciplinary Studies, Glendon College, York University, Toronto, Ontario, Canada; Universidad de Guadalajara, MEXICO

## Abstract

Relationships between parasites, host physiology, and behaviours are complex. Parasites can influence host hormonal microenvironment and behaviour through “sickness behaviours” that generally conserve energy. Using a parasite removal experiment, we examined the effects of gastrointestinal parasites on fecal glucocorticoid metabolites (fGC) and behaviours of vervet monkeys (*Chlorocebus pygerythrus*) at Lake Nabugabo, Uganda. We collected parasitological, hormonal, and behavioural data from adult and subadult male and female vervets (N = 19) in 2014 across four study phases: pre-deworming, post-deworming, early reinfection, and late reinfection as well as in 2015. Overall, there was no decrease in fGC after deworming, but there was an increase following natural reinfection. There was no change in feeding across study phases; however, moving, grooming, and resting changed between the post-deworming and late reinfection phases, but not always in the predicted direction. Comparing behaviour across the same months in the following year as in the 2014 experimental study period, we found no differences in moving, feeding, grooming, and resting events. Despite behavioural variation between study phases, we cannot conclude that behavioural changes are due to parasitism rather than other seasonal variation. However, fGC increased following reinfection, which is consistent with parasitism being costly for hosts.

## Introduction

Parasites live on or in a host organism, from whom they extract resources (e.g., nutrients) at the expense of their host [1]. Parasites can affect host health, behaviours, reproduction, survival, and overall fitness [1,2], and at the population level, can affect host density and distribution [1,3].

Although gastrointestinal parasites are less likely to be lethal to their hosts compared to viruses and bacteria, chronic infections from these parasites can lead to increased host morbidity [4], and induce various physiological and behavioural changes in their hosts by modulating and disrupting the immune system [5].

Organisms can respond physiologically and behaviourally to environmental or biological stressors [6]. One physiological response in vertebrates is the activation of the hypothalamic-pituitary-adrenal (HPA) axis, which leads to the release of glucocorticoid hormones like corticosterone and cortisol [7]. However, the relationships between host physiology and parasites are complex and ambiguous. Glucocorticoid hormones (GCs) increase temporarily in response to stressors and mobilize energy stores required to maintain homeostasis [8]. However, GCs can also have immunosuppressive effects leaving the hosts more susceptible to infections [9]. While a positive correlation between GCs and parasites may be due to HPA axis activation in response to parasite-imposed energetic stress [10], it is unclear whether the relationship is influenced by the resulting immunosuppression, the host’s stress response itself, or both [11,12]. Conversely, a negative relationship between GCs and parasites may arise from chronic stress causing HPA axis dysfunction [13], which can result in low GC levels [14]. Previous studies examining the link between parasites and GCs have shown mixed results, with some finding positive or negative relationships and others showing none; this variable relationship could be due to differences in factors such as parasite type, infection severity, and host age and sex [14]. A phylogenetic meta-analysis of 65 studies between parasites and mammalian host GCs revealed stronger support for a positive association than negative association. This could potentially be explained by a variety of mechanisms, including host manipulation by parasites, host responses to infection, cumulative stress effects, and the neuro-immunomodulatory functions of GCs [14].

Sickness behaviours are considered an adaptive response by the host’s immune system to fight infection and maintain homeostasis [15–17]. Sickness behaviours include general inactivity or lethargy, increased sleep, reduced exploratory behaviour, feeding, social, and sexual interactions, and postures that reduce heat loss and facilitate a fever response [18,19]. The species studied both in controlled and natural environments exhibit common sickness behaviors, such as anorexia, reduced nest building [20], decreased activity [21,22], less allogrooming [22], and increased somnolence [23] and resting [24]. Host sickness behaviours may also reduce contact with potentially infectious individuals by reducing movement and social grooming [25]. For example, studies have found that reduced movement in immune-challenged mice led to social disconnection [26], and *Trichuris*-infected vervet monkeys spent less time grooming conspecifics [27]. Alternatively, pathogens may directly influence host sickness behaviours that increase transmission opportunities, and in some cases may even alter the behaviour of uninfected individuals [28].

We used a parasite removal experiment and subsequent natural reinfection to examine the effects of gastrointestinal (GI) parasites on fGC and behaviours in a group of vervet monkeys (*Chlorocebus pygerythrus*) at Lake Nabugabo, Uganda. Previous research on this population found that it had greater parasite species richness than any other wild vervet population examined [29]. The Nabugabo vervet parasite community included trematodes such as *Dicrocoeliida* and *Schistosoma*, nematodes such as *Ascaris,* order Strongylida, and *Trichuris*, unidentified cestodes, protists such as *Entamoeba* and *Giardia*, and three parasite genera previously undocumented in vervets: *Metastrongylus, Toxocara,* and *Fasciola* [29]. Unidentified trematodes were the most prevalent, occurring in 92% of individuals at least once, followed by *Fasciola* spp. (38%), unidentified cestodes (38%), *Ascaris* spp. (33%), and *Strongyloides* spp. (29%) [29]. Experimental removal of GI parasites demonstrated resting events decreased and traveling events increased following deworming, as well as an increase in the number of nearest neighbors, especially for juveniles [30]. The current study expands on the previous study by examining fGC and behavioural response to deworming and reinfection, and behavioural variation was compared to a matched control phase the following year. We tested the hypothesis that if vervets respond physiologically to parasite infection, then we predict 1) a decrease in fGC after deworming and increase following reinfection. The logic of this prediction is that infection can activate the HPA, leading to GC secretion, as the host reallocates resources from non-essential functions to critical functions. If vervets respond behaviourally to parasite infection, we predict that 2) after deworming, there will be an increase in moving, feeding, and grooming behaviour and a decrease in resting but 3) a decrease in moving, feeding, and grooming and an increase in resting following natural reinfection.

## Methods

### Study site and subjects

The study was conducted on the shores of Lake Nabugabo, Uganda (0°22′–12°S, 31°54′E, 1,136 m), a satellite of Lake Victoria, that is mostly surrounded by wetlands, grasslands, patches of degraded forest, and farmers’ fields [30]. The area receives an average of 1348 mm of rain annually [30] in two seasons with high rainfall (March to May and September to November) and two seasons with low rainfall (December to February and June to August) [31].

During the experimental study (29 May – 13 December 2014), the group had an average of 28 individuals (5 adult males, 10 adult females, 1 subadult male, 2 subadult females, 10 juveniles and infants), each of whom was identifiable based on natural markings. Infants and juveniles were excluded from the experimental design. For our analyses, the category “male” includes adult (> 5 years) and subadult (4–5 years) males, “female with infant” includes females with infants < 6 months, and “female” includes all other adult (after first birth) and subadult (after first observed copulation) females.

### Study design

Parasitological, hormonal, and behavioural data were collected during all phases except the experimental deworming phase (see below). Subjects were dewormed to examine fecal glucocorticoid and behavioural changes in the presence and absence of gastrointestinal parasites.

***Pre-deworming phase*:** In the three weeks prior to experimental deworming treatment (29 May - 21 June 2014), we collected a mean of 4.2 fecal samples (range: 1–7) per individual for parasite and hormone analyses (see below). Daily behavioural activity scan sampling occurred from dawn to dusk every 30 minutes (see below).

***Deworming treatment*:** All subjects were treated twice using ivermectin at a dose of 0.3 mg/kg to kill helminth parasites, with a minimum of 5 days and a maximum of 7 days between treatments (treatment dates 22 June - 02 July 2014). Oral ivermectin was placed within a banana and opportunistically given to specific individuals when they were foraging in isolation from other individuals.

***Post-deworming phase*:** In the month following the ivermectin treatment (i.e., July 2014), we collected a mean of 3.4 fecal samples (range: 1–7) per individual and once again started activity scan sampling.

***Reinfection phases*:** Scan sampling was reduced to approximately 10 days per month for 8-hours per day during the early reinfection phase (August 2014) and late reinfection phase (September– December 2014). We collected 2 fecal samples per individual in the early reinfection phase and a mean of 3.8 fecal samples (range: 1–6) per individual in the late reinfection phase.

#### Parasitological data.

Fecal samples (N = 225, S1 Table) were collected immediately upon defecation and placed in vials labeled with individual, date, and time of collection, and then stored in a cooler with icepacks. At the end of the day, 1.0 g of wet fecal material was stored in 2 mL of 10% formalin solution for parasite identification, and approximately 0.1 g was placed in polyvinyl alcohol (PVA) for protozoan analysis. Parasites were identified to the family and genus level where possible at the Central Diagnostic Laboratory, College of Veterinary Medicine Animal Resources and Biosecurity, Makerere University, Kampala, Uganda using a modified ethyl acetate sedimentation method [32]. We calculated the prevalence of a parasite taxon by dividing the total number of individuals infected with a specific parasite taxon by the total number of individuals sampled. Maximum parasite species richness (MPSR) was tabulated from the unique number of parasite taxa found in each host’s fecal samples during each study phase [33].

#### Hormonal data.

We transferred ~ 1.0 g of wet feces from 182 (S1 Table) fecal samples to a labelled tube and stored these at −20°C until hormone extraction. Hormones were extracted from 0.5 g sample of thawed feces using a 10 mL solution of 50:50 deionized water: 95% ethanol, vortexed for 10 minutes, then centrifuged for 20 minutes. A 2 mL portion of the hormone extract was pushed through Prevail C18 Maxi-Clean 300mg Solid Phase Extraction cartridges (Alltech Associates, Inc., Deerfield, IL) and the cartridges were then washed with 2 mL of deionized water. Capped cartridges were stored in a cool, dark, and dry location before transport to McGill University for elution. SPE cartridges were washed with 1 mL of 5% methanol, and hormone extracts eluted from the cartridges with 2 mL of 100% methanol. Hormone-methanol extracts were stored at −4˚C before transport and fGC analyses [34].

Fecal cortisol metabolites were assayed by enzyme immunoassays (EIA) at the Toronto Zoo’s Reproductive Endocrinology Lab using a previously published protocol [35] with modification. Plates were coated with 50 ul of antibody (R4866; C. Munro, UC Davis) diluted 1:12,000 in coating buffer. After overnight incubation, plates were washed with 0.15 M NaCl and 0.05% Tween 20 and the wells loaded with 50 uL of hormone standards or reconstituted extracts followed by 50 uL of horseradish peroxidase conjugate diluted 1:33,400 in EIA buffer. For each sample, a 450 uL portion of the hormone-methanol extract was dried and resuspended in 150 uL assay buffer. To fit within the linear range of the standard curve, one sample was diluted 1:1 (150 ul evaporated extract, reconstituted in 150 ul buffer). After a two-hour incubation, plates were washed and 100 uL of colour developing substrate solution (i.e., ABTS) was added. After approximately 30 minutes of color development, absorbance was measured at 405 nm using a spectrophotometer (MRX microplate reader, Dynex Technologies, Chantilly, VA, USA). All samples and standards were run in duplicate, and all hormone values are reported in ng/g of wet fecal weight as a mean of the sample duplicates.

All assays included a 9-point standard curve (GC standard: Sigma H0135, 78–20,000 pg/mL) and two external fecal extract controls. The intra-assay CV was 7.4% at 55% binding, and inter-assay CVs were 9.7% and 6.4% for the low control (60% binding) and high control (19% binding), respectively. Assay sensitivity was 41.8 pg/ml. To test for parallelism between the standard curve and a representative sample pool, the pool was serially diluted two-fold from 1.25x (concentrated) to 1:13 in EIA buffer for fGC. Parallel displacement between the standard curve and serial dilutions of fecal extract was used as an indirect measure of assay specificity. Pooled reconstituted fecal extracts were serially diluted two-fold in assay buffer and visually compared to the respective standard curve. The data were plotted as log (relative dose) vs. percent antibody bound. The slopes of the lines within the linear portion of the curves were determined using linear regression analysis and compared (t = 0.69, p = 0.51, df = 7, S1 Fig) where p > 0.05 indicates the slopes are not significantly different and thus interpreted as parallel [36]. The relative standard deviation between samples in the dilution series was calculated using the method outlined by [37]. The %CV between neat and each dilution in series was ≤ 30% demonstrating parallelism [38].

Recovery of known amounts of cortisol was calculated to examine possible interference of fecal extract components with antibody binding. Increasing concentrations of hormone standard were added to an equal volume of pooled fecal extracts of known concentration. The percent recovery was calculated using the formula: amount observed/amount expected x 100%, where amount observed is the value from the spiked sample minus the endogenous hormone in the unspiked extract, and the amount expected is the concentration of hormone standard added. Recovery was mean ± SE = 92.6 ± 2.2% and the amount of cortisol recovered was correlated with the amount added (Pearson’s correlation coefficient (r) = 0.99, p < 0.001, S2 Fig). Fecal GC measured using a cortisol EIA have been previously validated for biological relevance in captive vervets as evidenced by an fGC increase in response to an ACTH challenge [39].

#### Behavioural data.

The occurrence of general activities, such as moving, feeding, resting, grooming (both receiving and giving), other affiliative behaviours (playing, groom solicit, etc.), agonism (chase, avoid, supplant), and mating behaviours (copulations, mating presentations) were recorded every 30 minutes following an established ethogram (S1 Ethogram). At each behavioural activity scan sample, the behaviour and nearest neighbor were recorded for five subjects. All subjects were sampled at least once a day (S1 Table). Data were collected by 2 observers. The observers went through extensive training to ensure consistency in how they recorded behaviours and to limit interobserver variation. Given this training and since the behaviours are easy to distinguish between (e.g., resting versus moving) we believe there was little interobserver variation in how behaviour was assessed.

### Statistics

We analyzed the effect of maximum parasite species richness on fGC using linear mixed effects models (LMMs). The vervets continued to excrete parasites in their feces shortly after deworming, likely consisting of inactive/dead parasites. Considering that the typical life cycles and prepatent periods of the identified nematodes, trematodes, and cestodes ranges from 14 to 44 days [40,41], we inferred a delay of approximately one month between the parasite presence in feces and on-going gastrointestinal infection, and thus compared excreted fecal parasite species richness in one month to fecal glucocorticoid levels (fGC) in the previous month. This predictor variable is herein referred to as “lagged MPSR” (e.g., MPSR in August was examined in relation to July fGC levels). For the LMM, we created a repeated-measures global model with fGC levels as the response variable and lagged MPSR (0, 1, ≥ 2 taxa), study phase (4 phases: pre-deworming, post-deworming, early reinfection, and late reinfection), sex/reproductive state (male, female without infant, and female with infant) as fixed effects; we included individual identity as a random effect. Although the goal of the study is not to examine sex/reproductive status differences, we included it as a predictor variable because it is a possible source of variation that we can account for since females may have different baseline GC levels than males due to the energetic demands associated gestation and lactation [42]. Since some of the females gave birth during the study, we occasionally characterised the same individual as adult female without infant (pre-parturition) and female with infant (post-partum) within the same phase (N = 2).

Activity budgets were calculated from 4710 scans in 2014 and 5330 in 2015 for males, females, and females with infants. The number of study individuals changed between 2014 and 2015 due to demographic changes (S2 Table). We divided general activity into four categories: moving, feeding, social grooming (i.e., giving and receiving grooming), and resting. Generalized linear mixed effects models (GLMMs) with binomial link function (package lmerTest) [43] were used to examine the effect of lagged MPSR on moving, feeding, grooming, and resting activity scans. For each behaviour, we created a repeated-measures global model with lagged MPSR (0, 1, ≥ 2 taxa), study phase (4 phases: pre-deworming, post-deworming, early reinfection, and late reinfection), mean individual monthly fGC, and sex/reproductive status (male, female without infant, and female with infant) as fixed effects and vervet identity as a random effect. We used the total number of activity scans per individual per phase as weights to account for the variable contribution of individuals to the dataset [44]. If a female gave birth during a study phase, we characterised the individual as “female without infant” if the number of days of activity scan data collection before the birth exceeded the number of days of data collection after the birth and characterised the individual as “female with infant” if the reverse was true [40]. For each model, we used the ‘dredge’ function in the Multi-model Inference (MuMIN) package [45] to identify the “top” models within Δ7 AICc [46]. When multiple top models were identified, we conducted model averaging and calculated 95% confidence interval for each predictor [46,47].

To examine whether the behavioural changes in 2014 were associated with the deworming experiment rather than naturally-occurring social (e.g., conceptive vs. birth peaks) or ecological (e.g., rain and temperature variation) seasonal variations, we compared behaviour categories (i.e., proportion of activity scans for moving, feeding, grooming, and resting) across months in 2015 (non-experimental period) using separate generalized linear models (GLMs) with month, and sex/reproductive status as predictor variables. The total activity scans per individual per month were used as weights to account for the variable contribution of individuals to the dataset in each period [44]. Despite potential effects of social (i.e., conception or birth “peaks”) and ecological season on hormones and behaviour, we did not include these in the model because births can and did occur outside of the birth “peak”, and the ecological seasonality (wet and “wetter” seasons) both occur in the late-reinfection phase. In order to avoid overfitting and unwarranted model complexity, and to focus on the relevant predictor variables, we did not include any interactions in our models. All statistical analyses were conducted in R statistical software RStudio (version 4.0.3) [48].

### Ethical note

This research was approved by the Uganda Wildlife Authority and Uganda National Committee for Science and Technology to CAC (2011–2015) and VAMS (2015-present), as well as the McGill University Animal Care Committee to CAC and York University Animal Care Committee to VAMS.

### Inclusivity in global research

Additional information regarding the ethical, cultural, and scientific considerations specific to inclusivity in global research is included in the Supporting information (S1 Checklist).

## Results

### Parasites and study phases

We collected 225 fecal samples to identify gastrointestinal parasites during the 2014 experimental study phases, with a mean ± SE = 0.97 ± 0.07 (range = 0–4, median = 1), of which 154 (68.4%) were positive for at least one parasite taxon. Of the 154 positive samples, 62.9% were positive for a single parasite taxon, while 33.1% were positive for two taxa, 2.6% for three taxa, and 1.3% for four taxa. The most commonly found parasites were unidentified trematodes, followed by unidentified cestodes and coccidian oocysts (S3 Table). *Schistosoma* spp. was the only identified trematode, no cestode taxa were identified, and *Strongyloides* spp. were found to be most prevalent nematodes (S3 Table). Among protists, *Iodomoeba* spp. were the most prevalent (S3 Table). Of the 72 fecal samples collected during the pre-deworming phase, 81% were positive for at least one parasite (S3 Fig). Following deworming and reinfection, 87% of fecal samples collected (n = 52) were infected, and all parasite taxa were present. Consistent with the average life cycles and prepatent periods of known vervet parasites, we found that: 1) the first month of reinfection (i.e., August) was marked by the absence of shed trematodes, cestodes, or nematodes (n = 32), with only 9% of samples testing positive for protists, and 2) the second month of reinfection, 100% samples were positive for parasites, with 87% for trematodes, 57% for cestodes, 0% for nematodes, and 9% for protists (S3 Fig).

### Effect of gastrointestinal parasites on fecal glucocorticoids

**Fig 1:**
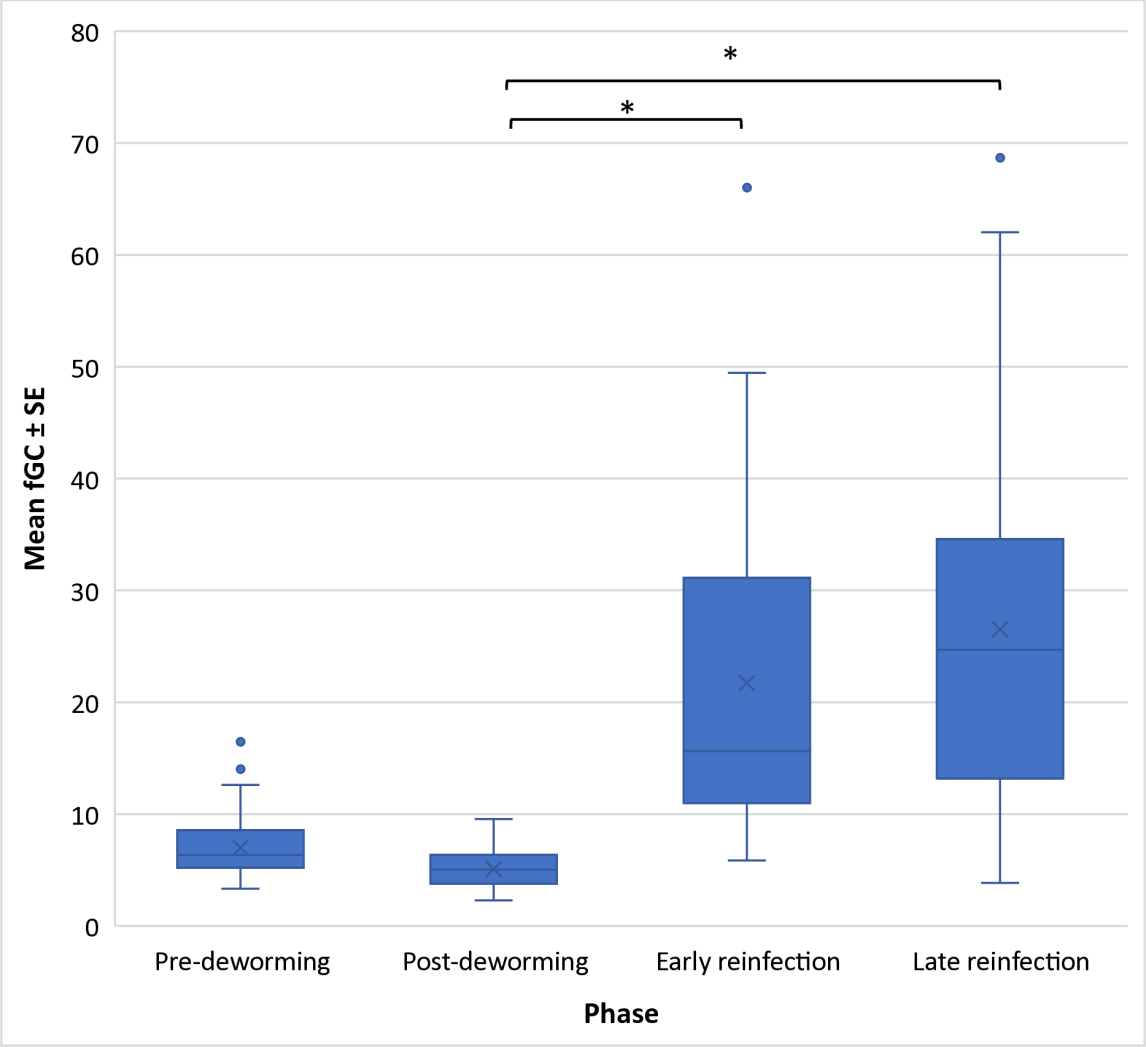
Fecal glucocorticoid (fGC) metabolite levels in vervet monkeys across study phases. The estimated mean fGC levels (X) in vervet monkeys during each study phase at Lake Nabugabo, Uganda, from June-December 2014. * Significant pairwise comparison.

Across the experimental study period, the mean ± SE fGC was 16.51 ng/g ± 1.71 ng/g (median: 8.62 ng/g, range: 2.29 – 297.49 ng/g; S1 Table). Changes in mean fGC were best described by three top models, all of which included study phase, while lagged MPSR and sex/reproductive state each appeared in two models (Table 1). Contrary to our prediction, mean fGC did not decrease from pre- to post-deworming. As predicted, fGC levels were higher during the reinfection phases [both early (b = 13.07, SE = 3.67, CI: 5.83 – 20.30) and late (b = 19.74, SE = 2.47, CI: 14.86 – 24.61)] than during the post-deworming phase (Fig 1; S4 Table).

**Table 1:** Model selection for the outcome variable fecal glucocorticoid metabolites (fGC) during deworming experimental year (2014) in vervet monkeys (*Chlorocebus pygerythrus*) at Lake Nabugabo, Uganda.

Outcome	Predictors	df	loglik	AICc	ΔAICc	weight
fGC	Phase + Lagged MPSR + Sex	9	−660.37	1339.80	0	0.75
	Phase + Lagged MPSR	7	−664.27	1343.20	3.39	0.14
	Phase + Sex	8	−663.51	1343.90	4.06	0.09

Predictors include study phase (pre-deworming, post-deworming, early reinfection, late reinfection), lagged maximum parasite species richness (lagged MPSR), and sex (sex/reproductive state = males, females without infants, and females with infants). The models are within < 7 ΔAICc.

### Effects of gastrointestinal parasites on behaviours

On average, the proportion of moving, feeding, grooming, and resting activity scans were 17%, 30%, 16%, and 16% respectively from June to December 2014 (S2 Table), which is similar to the proportion of moving, feeding, grooming, and resting activity scans of 17%, 34%, 13%, and 15% respectively in June to December 2015 (S2 Table).

#### Moving.

**Fig 2:**
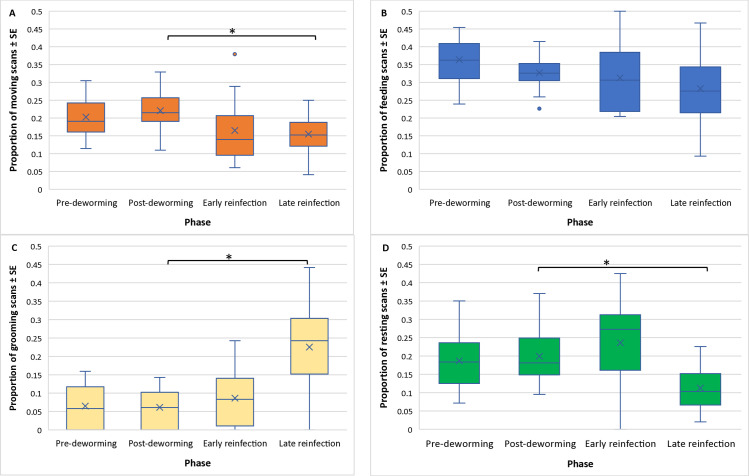
The proportion of (A) moving, (B) feeding, (C) grooming, and (D) resting activity scans in vervet monkeys across study phases. The estimated mean proportion (X) of moving, feeding, grooming, and resting activity scans in vervet monkeys (*Chlorocebus pygerythrus*) during each study phase at Lake Nabugabo, Uganda, from June-December 2014. * Significant pairwise comparison.

Variation in moving was best described by eight top models, all of which included phase, while lagged MPSR, mean fGC, and sex/reproductive state each appeared in four models (Table 2). Moving was lower in the late reinfection phase compared to the post-deworming phase (b = −0.34, SE = 0.13, CI: −0.58 to −0.08) (S5 Table; Fig 2A), but there were no other differences between study phases. Moving behaviour did not differ across months in 2015 (S6 Table).

**Table 2:** Model selection for the behavioural outcome variables during experimental deworming year in vervet monkeys (*Chlorocebus pygerythrus*) at Lake Nabugabo, Uganda.

Outcomes	Predictors	df	loglik	AICc	ΔAICc	weight
Proportion of moving scans	Phase + Lagged MPSR	6	−203.39	419.90	0	0.37
	Phase + Lagged MPSR + Mean fGC	7	−202.78	421.10	1.17	0.21
	Phase + Lagged MPSR + Sex	8	−201.69	421.40	1.45	0.18
	Phase	5	−205.91	422.60	2.69	0.09
	Phase + Lagged MPSR + Mean fGC + Sex	9	−201.25	423.00	3.09	0.08
	Phase + Sex	7	−204.08	423.70	3.76	0.06
	Phase + Mean fGC	6	−205.39	423.89	3.98	0.05
	Phase + Mean fGC + Sex	8	−203.77	425.51	5.60	0.02
Proportion of feeding scans	Phase + Sex	7	−225.92	467.35	0	0.28
	Phase + Mean fGC + Sex	8	−224.98	467.93	0.58	0.21
	Mean fGC + Sex	5	−228.61	468.01	0.65	0.20
	Mean fGC + Lagged MPSR + Sex	6	−227.67	468.46	1.11	0.16
	Phase + Lagged MPSR + Sex	8	−225.92	469.80	2.45	0.08
	Phase + Mean fGC + Lagged MPSR + Sex	9	−224.98	470.45	3.10	0.06
Proportion of grooming scans	Phase + Sex	7	−200.56	416.60	0	0.45
	Phase + Lagged MPSR + Sex	8	−199.72	417.40	0.76	0.31
	Phase + Mean fGC + Sex	8	−200.44	418.90	2.21	0.19
	Phase + Mean fGC + Lagged MPSR + Sex	9	−199.58	419.70	3.02	0.09
Proportion of resting scans	Phase	5	−234.47	479.74	0	0.33
	Phase + Lagged MPSR	6	−233.81	480.75	1.01	0.20
	Phase + Mean fGC	6	−233.88	480.89	1.15	0.18
	Phase + Mean fGC + Lagged MPSR	7	−233.16	481.82	2.09	0.12
	Phase + Sex	7	−233.51	482.52	2.79	0.08
	Phase + Mean fGC + Sex	8	−233.05	484.07	4.34	0.04
	Phase + Lagged MPSR + Sex	8	−233.05	484.08	4.34	0.04
	Phase + Mean fGC + Lagged MPSR + Sex	9	−232.53	485.56	5.82	0.02

Predictors include study phase, lagged maximum parasite species richness (lagged MPSR), mean fGC, and sex. The models are within < 7 ΔAICc.

#### Feeding.

Variation in feeding was best described by six top models, with the variable sex/reproductive state appearing in all six models, phase and mean fGC appearing in four models, and lagged MPSR appearing in three models (Table 2). There was no difference in feeding behaviour across phases (S5 Table; Fig 2B), but females with infants fed less than females without infants (b = −0.38, SE = 0.09, CI: −0.56 to −0.18) (S5 Table). Similarly, there was no variation in feeding behaviour across months in 2015 (S6 Table).

#### Grooming.

Variation in grooming was best described by four top models, all of which included phase and sex/reproductive state, with lagged MPSR and mean fGC each appearing in two top models (Table 2). Grooming was higher in the late reinfection phase compared to the post-deworming phase (b = 1.39, SE = 0.15, CI: 1.11 to 1.69) (S5 Table; Fig 2C), but there were no other differences between study phases. Additionally, males spent less time grooming than females without infants (b = −2.44, SE = 0.34, CI: −3.13 to −1.75) (S5 Table; Fig 2C). There was no variation in grooming behaviour throughout 2015 (S6 Table).

#### Resting.

Variation in resting was best described by eight top models, all of which included phase, with lagged MPSR, mean fGC, and sex/reproductive state each appearing in four of the top models (Table 2). Resting was lower in late reinfection phase (b = −0.71, SE = 0.13, CI: −0.96 to −0.45) (S5 Table; Fig 2D), but there were no other differences between study phases. There was no variation in resting behaviour throughout 2015 (S6 Table).

## Discussion

We examined changes in fecal glucocorticoids and behaviour of vervet monkeys in response to an experimental ivermectin deworming treatment and natural reinfection. Contrary to our predictions, there was no decrease in fecal glucocorticoids, nor were there any behavioural changes in response to deworming. However, fGCs increased following reinfection. Our behavioural analyses provide limited support for the presence of sickness behaviours in response to reinfection, with a decrease in moving in the late reinfection phase as predicted. However, in contrast to our predictions, there was no change in feeding behaviour, while following reinfection grooming and resting changed in the opposite direction from what we had predicted (an increase in grooming, a decrease in resting). Furthermore, there were differences in behaviour across the experimental phases but not in the non-experimental year, suggesting that vervets adjust their behaviour in response to parasite infection.

### Effectiveness of ivermectin treatment

Species richness of parasites shed in vervet feces decreased in the second month after deworming. This is consistent with a study where silver foxes were treated with ivermectin, resulting in parasite-free fecal samples 3- to 6-weeks post-treatment [49]. The “delayed” decrease in excreted parasites following ivermectin treatment may be because the vervets were shedding inert parasites during the first month after deworming. Given that ivermectin is an anti-helminthic treatment, it is not surprising that only protists were shed in the second month after deworming.

### Effects of parasites on fecal glucocorticoids

Contrary to our prediction, mean fecal glucocorticoids (fGC) did not decrease following ivermectin treatment, but it did increase following parasite reinfection. The absence of a decrease in GC may be the result of the fact that a parasite may have diminished effects on a host if the host’s tolerance to the damage caused by the parasite is chronic [50] and/or it may suggest they use compensatory behavioural mechanisms, such as sickness behaviours (but see below) to limit the detrimental effects of parasite infection. Our findings are consistent with those of a parasite reduction experiment in racoons that also found no decrease in fecal glucocorticoids [51]. Parasite infected reindeer had slightly lower glucocorticoid levels than uninfected reindeer, leading to authors to hypothesize that they may use a tolerance strategy to cope with infection [52]. Another possible explanation for the lack of GC decrease following deworming may be due to individual variation in the impact of parasitism, influenced by factors such as age, sex, reproductive state, dominance status, or sociality [1,53], which we were unable to examine since model averaging makes the interpretation of interactions very difficult [54]. However, if parasites are indeed costly, it is difficult to imagine a scenario where an individual would not benefit from deworming; that said, individual characteristics (e.g., dominance rank) may influence the occurrence and response to other (non-parasite) stressors [55].

As predicted, there was an elevation in fGC levels as the vervets became reinfected. This may reflect a stress response to reinfection and aligns with the idea that short-term elevations in GCs are adaptive due to their anti-inflammatory effects as a part of the immune reaction [7,56]. This has been observed in mice where initial parasite infection was associated with elevations in blood glucocorticoids [57]. Possibly the process of deworming disrupted any homeostatic state that may have been reached by hosts and that a new infection is more likely to illicit a response than an on-going, low-grade infection. A diminished or incomplete immune response has been observed in rats infected with *Nippostrongylus brasiliensis*, with immune response returning to normal following antihelminthic treatment [58]. However, both hosts and parasites can influence GC levels when parasite infection occurs [14]. Some parasites manipulate GC levels, which can increase susceptibility to additional infections [59]. Some parasites may also be associated with secondary infections by other parasites [60] by causing immunosuppression due to long-term elevation of GCs [9]. This could explain the observed increase in fGC levels during the early and late reinfection phase. Nevertheless, other factors could have influenced the increase, along with parasite reinfection. For example, glucocorticoids are known to vary with social interactions as well as with social and ecological seasonality [61].

### Effects of parasites on behaviours

Contrary to our predictions, there was no evidence of sickness behaviours that changed the balance of moving, feeding, grooming, or resting in response to experimental deworming. However, we observed changes in moving, grooming, and resting between the post-deworming and late reinfection phase, though the direction of these behavioural changes were only partially consistent with the hypothesis that parasite infection can lead to reduced energy budgets [18]. Consistent with our prediction, movement, an energetically expensive behaviour, was lower in the late reinfection phase. This reduced movement could be a strategy to preserve energy while mounting an immune response, which is consistent with our findings that fGCs also increased at this time. A decrease in moving behaviour could also be a host strategy to decrease the chances of encountering parasites in the environment when they are already fighting an existing parasite infection [62,63].

In contrast to our predictions, resting decreased while grooming increased during the reinfection phase, and feeding behaviour did not change between study phases. We had expected resting, an energetically conservative behaviour, to increase during the reinfection phase compared to the post-deworming phase. We had also expected that grooming and feeding, energetically expensive behaviours and one that also have the potential to increase parasite exposure risk, would decrease during the reinfection phase [27,63]. Grooming is an important behaviour in developing and maintaining social bonds but is also a source of disease transmission [64]. One study found that parasite-infected Barbary macaques (*Macaca sylvanus*) continued to maintain social interactions despite elevated glucocorticoids, a finding which highlights the importance of social relationships [65].

The observed decrease in moving and resting behaviour and increase in grooming during the late reinfection phase could be driven by a subset of individuals: females with infants. This period coincides with a birth peak in the Nabugabo vervet population, such that females with infants may be constrained by parental care, possibly including increased grooming towards infants and decreased moving (such as during breastfeeding) and therefore have less time for other activities. In our study, five females gave birth during the late reinfection phase, compared to two in the early reinfection phase and one during the post-deworming phase. That said, this seems unlikely given that behaviour did not vary across 2015 despite the birth of three infants during late reinfection phase, although we did find that females with infants fed less than females without infants in the experimental year. Increased grooming may be associated with the birth season, when not only mothers but many individuals are drawn to new infants and access to them may be used as a commodity in exchange for grooming [66]. Unfortunately, we were not able to test this hypothesis because we would need to incorporate interactions into our models, which can complicate interpretation and lead to falsely interpreting biologically significant interactions as statistically non-significant when model averaging [54].

Another possible alternative explanation for changes in moving, resting, and grooming across the experimental year could be ecological seasonality. Despite the occurrence of two wet and two dry seasons at the Lake Nabugabo field site, there is no seasonality in the availability of the most consumed natural foods, nor in the consumption of anthropogenic foods. Furthermore, we did not find any behavioural variation during the corresponding time periods in the non-experimental year. Another limitation of model averaging that we need to keep in mind is that it focuses on prediction rather than causality. While it excels at identifying a set of predictors that best explain the variation, it does not provide insight into the underlying mechanisms or causal pathways. Thus, some observed behavioural changes were consistent with sickness behaviour-related changes (i.e., decreased moving during late reinfection), while others were not (i.e., increase in grooming and decrease in resting).

Overall, our ivermectin treatment experiment provided mixed support of the hypothesis that gastrointestinal parasites are costly for hosts. Deworming had no impact on fGC levels or any of the behaviours examined, which suggests that helminth parasites may not be costly to hosts or that infections are sufficiently low-grade that parasite removal has a limited impact. The fact that behaviours did not change in response to the deworming, suggesting that they do not appear to use compensatory behavioural mechanisms to mitigate costs of parasite infection. Another possible explanation for our findings is the “old friends” derivative of the “hygiene hypothesis”, which suggests that because parasitic helminths have co-evolved with the immune system, they play a crucial role in supporting normal immune development [67,68]. Thus, it is possible that these parasites may contribute to maintaining a healthy immune response rather than being solely detrimental in the vervets. Additionally, the pathogenicity of the identified parasite taxa could influence the results, as previous research has shown that only certain parasites are associated with elevated fGC levels [53]. That said, there was an increase in fGC following natural reinfection and a decrease in movement behaviour, as would be predicted if parasites are costly for hosts. Although we cannot exclude the possibility that the observed fGC and behavioural variation is tied to seasonality rather than parasitism, it is possible that gastrointestinal parasites are not as costly as previously hypothesized or host physiological responses to parasitism are sufficient to limit the expression of sickness behaviours.

## Supporting information

S1 Table Parasite and fecal glucocorticoid metabolites (fGC) data. Summary of parasite data and number of samples assayed for fGC from fecal samples collected from 19 adults in one group of vervet monkeys (*Chlorocebus pygerythrus*) at Lake Nabugabo, Uganda between June-December 2014. * AFI = Adult female with infant.
(DOCX)

S2 Table Percentage of scans spent in each category of behaviours by males, females, and females with infants of vervet monkeys at Lake Nabugabo, Uganda, from June-December 2014 and 2015. * AFI = Adult female with infant.
(DOCX)

S3 Table Parasite taxa, number of parasites found, and prevalence of parasite. Summary of parasite taxa, and their prevalence from 19 adult vervet monkeys (*Chlorocebus pygerythrus*) at Lake Nabugabo, Uganda from June-December 2014. Prevalence is calculated as the number of individuals infected with a given parasite taxon, divided by the total number of individuals sampled.
(DOCX)

S4 Table Model averaged parameter estimates for the top models for mean fecal glucocorticoid metabolites (fGC) outcome. The table shows relative importance (∑), regression coefficient (*b*), unconditional standard error (SE), and 95% confidence interval (CI) for *b*. Statistically significant predictors are in bold.
(DOCX)

S5 Table Model averaged parameter estimates for the top models for behavioral outcomes. The table contains relative importance (∑), regression coefficient (b), Unconditional standard error (SE) and 95% confidence interval (CI) for b. Statistically significant predictors are in bold.
(DOCX)

S6 Table Results of ANOVA following Generalized linear models (GLMs) for the behavioural outcome variables across months during the non-experimental year (2015) in vervet monkeys (*Chlorocebus pygerythrus*) at Lake Nabugabo, Uganda. Predictors include month (June – December), and sex. The outcome variables include proportion of moving, feeding, grooming, and resting scans.
(DOCX)

S1 Fig Vervet monkey (*Chlorocebus pygerythrus*) fecal cortisol parallelism (t = 0.69, p = 0.51, df = 7). (TIF)

S2 Fig Recovery of standard hormone added to a pooled fecal extract and the amount of cortisol recovered was correlated with the amount added. (TIF)

S3 Fig Proportion of fecal samples infected with parasites. Proportion of infected samples with different parasite taxa (trematodes, cestodes, nematodes, & protists) during each study phase at Lake Nabugabo, Uganda, from June – December 2014.
(TIF)

S1 Ethogram Nabugabo vervet ethogram for activity data. (DOCX)

S1 Checklist (DOCX)
