# Supplementary material for: Effects of gastrointestinal parasites on fecal glucocorticoids and behaviour in vervet monkeys (Chlorocebus pygerythrus)
Source: PLoS One. 2025 Jan 30;20(1):e0316728. doi: 10.1371/journal.pone.0316728 (PMC11781662; doi:10.1371/journal.pone.0316728)
Supplement: S1 Table — Summary of parasite data and number of samples assayed for fGC from fecal samples collected from 19 adults in one group of vervet monkeys (Chlorocebus pygerythrus) at Lake Nabugabo, Uganda between June-December 2014. * AFI = Adult female with infant. (DOCX) [file pone.0316728.s001.docx]

**S1 Table.** **Parasite and fecal glucocorticoid metabolites (fGC) data**. Summary of parasite data and number of samples assayed for fGC from fecal samples collected from 19 adults in one group of vervet monkeys (Chlorocebus pygerythrus) at Lake Nabugabo, Uganda between June-December 2014. *AFI = Adult female with infant

| **Phase** | **Month** | **Sex** | **No. of individuals** | **% infected individuals** | **No. of samples** | **No. of positive samples** | **%**  **positive samples** | | **No. of samples assayed for fGC** |
| --- | --- | --- | --- | --- | --- | --- | --- | --- | --- |
| Pre-deworming | June | Male | 6 | 100% | 29 | 23 | 79% | 80.55% | 22 |
|  |  | Female | 10 | 100% | 37 | 29 | 78% |  | 37 |
|  |  | AFI* | 1 | 100% | 6 | 6 | 100% |  | 6 |
| Post-deworming | July | Male | 4 | 100% | 11 | 10 | 91% | 88.23% | 7 |
|  |  | Female | 10 | 100% | 31 | 27 | 87% |  | 23 |
|  |  | AFI* | 2 | 100% | 9 | 8 | 89% |  | 7 |
| Early reinfection | August | Male | 5 | 20% | 10 | 1 | 10% | 9.38% | 6 |
|  |  | Female | 10 | 10% | 19 | 2 | 11% |  | 16 |
|  |  | AFI* | 2 | 0% | 3 | 0 | 0% |  | 2 |
| Late reinfection | September | Male | 3 | 100% | 5 | 5 | 100% | 100% | 2 |
|  |  | Female | 8 | 100% | 14 | 14 | 100% |  | 14 |
|  |  | AFI* | 2 | 100% | 4 | 4 | 100% |  | 2 |
|  | October | Male | 2 | 100% | 3 | 2 | 67% | 45.45% | 2 |
|  |  | Female | 9 | 33% | 13 | 4 | 31% |  | 10 |
|  |  | AFI* | 3 | 100% | 6 | 4 | 67% |  | 3 |
|  | November | Male | 3 | 0% | 3 | 0 | 0% | 25% | 2 |
|  |  | Female | 6 | 50% | 6 | 3 | 50% |  | 5 |
|  |  | AFI* | 3 | 0% | 3 | 0 | 0% |  | 3 |
|  | December | Male | 2 | 100% | 2 | 2 | 100% | 92.31% | 1 |
|  |  | Female | 4 | 75% | 4 | 3 | 75% |  | 4 |
|  |  | AFI* | 7 | 100% | 7 | 7 | 100% |  | 6 |
|  | **Total** |  |  |  | **225** | **154** |  |  | **180** |
