# Supplementary material for: Effects of gastrointestinal parasites on fecal glucocorticoids and behaviour in vervet monkeys (Chlorocebus pygerythrus)
Source: PLoS One. 2025 Jan 30;20(1):e0316728. doi: 10.1371/journal.pone.0316728 (PMC11781662; doi:10.1371/journal.pone.0316728)
Supplement: S2 Table — * AFI = Adult female with infant. (DOCX) [file pone.0316728.s002.docx]

**S2 Table**: Percentage of scans spent in each category of behaviours by males, females, and females with infants of vervet monkeys at Lake Nabugabo, Uganda, from June-December 2014 and 2015. *AFI = Adult female with infant.

| **Year** | **Phase** | **Sex** | **No. of scans** | **Total no. of scans** | **Moving** | **Feeding** | **Grooming** | **Resting** |
| --- | --- | --- | --- | --- | --- | --- | --- | --- |
| 2014 | Pre-deworming (June) | Male | 293 | 1178 | 20% | 36% | 6% | 20% |
|  |  | Female | 791 |  |  |  |  |  |
|  |  | AFI* | 94 |  |  |  |  |  |
| 2014 | Post-deworming (July) | Male | 335 | 1431 | 22% | 32% | 6% | 20% |
|  |  | Female | 880 |  |  |  |  |  |
|  |  | AFI* | 216 |  |  |  |  |  |
| 2014 | Early reinfection (Aug.) | Male | 125 | 535 | 16% | 31% | 8% | 24% |
|  |  | Female | 321 |  |  |  |  |  |
|  |  | AFI* | 89 |  |  |  |  |  |
| 2014 | Late reinfection (Sept. - Dec.) | Male | 183 | 692 | 18% | 33% | 14% | 16% |
|  |  | Female | 883 |  |  |  |  |  |
|  |  | AFI* | 458 |  |  |  |  |  |
| 2015 | June | Male | 220 | 854 | 19% | 30% | 13% | 15% |
|  |  | Female | 466 |  |  |  |  |  |
|  |  | AFI* | 168 |  |  |  |  |  |
| 2015 | July | Male | 252 | 852 | 18% | 33% | 12% | 14% |
|  |  | Female | 438 |  |  |  |  |  |
|  |  | AFI* | 162 |  |  |  |  |  |
| 2015 | Aug. | Male | 219 | 692 | 18% | 33% | 14% | 16% |
|  |  | Female | 345 |  |  |  |  |  |
|  |  | AFI* | 128 |  |  |  |  |  |
| 2015 | Sept. - Dec. | Male | 1017 | 2932 | 16% | 35% | 13% | 15% |
|  |  | Female | 1439 |  |  |  |  |  |
|  |  | AFI* | 476 |  |  |  |  |  |
