# Supplementary material for: Effects of gastrointestinal parasites on fecal glucocorticoids and behaviour in vervet monkeys (Chlorocebus pygerythrus)
Source: PLoS One. 2025 Jan 30;20(1):e0316728. doi: 10.1371/journal.pone.0316728 (PMC11781662; doi:10.1371/journal.pone.0316728)
Supplement: S3 Table — Summary of parasite taxa, and their prevalence from 19 adult vervet monkeys (Chlorocebus pygerythrus) at Lake Nabugabo, Uganda from June-December 2014. Prevalence is calculated as the number of individuals infected with a given parasite taxon, divided by the total number of individuals sampled. (DOCX) [file pone.0316728.s003.docx]

**S3 Table.** **Parasite taxa, number of parasites found, and prevalence of parasite**. Summary of parasite taxa, and their prevalence from 19 adult vervet monkeys (*Chlorocebus pygerythrus*) at Lake Nabugabo, Uganda from June-December 2014. Prevalence is calculated as the number of individuals infected with a given parasite taxon, divided by the total number of individuals sampled.

| **Parasites** | **Taxon** | **No. positive samples (individuals)** | **Prevalence** | **Route of transmission** |
| --- | --- | --- | --- | --- |
| Trematodes | *Schistosoma* spp. | 1(1) | 5.3% | Direct contact^1^ |
|  | Trematode (unidentified) | 125(17) | 89.5% | - |
| Cestodes | Cestode (unidentified) | 17 (13) | 68.4% | - |
| Nematodes | *Ascaris* spp. | 1 (1) | 5.3% | Feco-oral route^2^ |
|  | *Strongyloides* spp. | 5 (5) | 26.3% | Soil^3^ |
|  | Strongyle | 2 (2) | 10.5% | Soil^4^ |
|  | *Trichostrongylus* spp. | 2 (2) | 10.5% | Feco-oral route^5^ |
|  | *Trichuris* spp. | 2 (2) | 10.5% | Oral route^5^ |
| Protists | *Amoeba* spp*.* | 5 (5) | 26.3% | - |
|  | Coccidian oocysts | 16 (10) | 52.6% | - |
|  | *Cryptosporidium* spp. | 2 (2) | 10.5% | Feco-oral route and direct contact^6^ |
|  | *Entamoeba coli* | 13 (8) | 42.1% | Oral route^5^ |
|  | *Entamoeba hartmanni* | 1 (1) | 5.3% | Oral route^5^ |
|  | *Entamoeba histolytica* | 5 (5) | 26.3% | Oral route^5^ |
|  | *Giardia* spp. | 1 (1) | 5.3% | Feco-oral route^7^ |
|  | *Iodamoeba* spp. | 16 (7) | 36.8% | Feco-oral route^8^ |
|  | Unidentified | 3 (3) | 15.8% | - |
| [1] Corachan. 2002. Schistosomiasis and International Travel. *Clin Infect Dis* 35: 446-50. [2] Asaolu & Ofoezie. 2019. Ascaris spp. In: Water and Sanitation for the 21st Century: Health and Microbiological Aspects of Excreta and Wastewater Management (Global Water Pathogen Project). Michigan State University. [3] Hasegawa et al. 2016. Strongyloides infections of humans and great apes in Dzanga-Sangha Protected Areas, Central African Republic and in degraded forest fragments in Bulindi, Uganda. *Parasitol Int* 65:367–70. [4] Ghai et al. 2014. Nodule Worm Infection in Humans and Wild Primates in Uganda: Cryptic Species in a Newly Identified Region of Human Transmission. *PLoS Negl Trop Dis* 8:39. [5] Boundenga et al. 2018. Diversity and prevalence of gastrointestinal parasites in two wild Galago species in Gabon. *Infect Genet Evol*. 63:249–56. [6] Feng et al. 2018 Genetic Diversity and Population Structure of Cryptosporidium. *Trends in Parasitol* 38: 997–1011. [7] Kramer et al. 2009 Treatment of Giardiasis in Common Marmosets (*Callithrix jacchus*) with Tinidazole. *Comp Med* 59:174–9. [8] Issa RM. 2014 Non-pathogenic protozoa. *Int J Pharm Pharm Sci 6* (suppl 40):30-40. | | | | |
