# Supplementary material for: Effects of gastrointestinal parasites on fecal glucocorticoids and behaviour in vervet monkeys (Chlorocebus pygerythrus)
Source: PLoS One. 2025 Jan 30;20(1):e0316728. doi: 10.1371/journal.pone.0316728 (PMC11781662; doi:10.1371/journal.pone.0316728)
Supplement: S4 Table — The table shows relative importance (∑), regression coefficient (b), unconditional standard error (SE), and 95% confidence interval (CI) for b. Statistically significant predictors are in bold. (DOCX) [file pone.0316728.s004.docx]

**S4 Table.** **Model averaged parameter estimates for the top models for mean fecal glucocorticoid metabolites (fGC) outcome**. The table shows relative importance (∑), regression coefficient (*b*), unconditional standard error (SE), and 95% confidence interval (CI) for *b*. Statistically significant predictors are in bold.

| **Outcome** | **Predictors/Levels** | **∑** | ***b*** | **SE** | **95% CI for *b*** |
| --- | --- | --- | --- | --- | --- |
| fGC | Phase_Pre-deworming | 1.00 | -1.71 | 3.33 | -8.28 to 4.86 |
|  | **Phase_Early reinfection** |  | **13.07** | **3.67** | **5.83 to 20.30** |
|  | **Phase_Late reinfection** |  | **19.74** | **2.47** | **14.86 to 24.61** |
|  | Lagged MPSR | 0.90 | 2.58 | 1.64 | -0.67 to 5.82 |
|  | Sex_Male | 0.86 | -1.23 | 2.17 | -5.52 to 3.05 |
|  | Sex_AFI |  | 0.98 | 2.19 | -3.36 to 5.32 |
