# Supplementary material for: Effects of gastrointestinal parasites on fecal glucocorticoids and behaviour in vervet monkeys (Chlorocebus pygerythrus)
Source: PLoS One. 2025 Jan 30;20(1):e0316728. doi: 10.1371/journal.pone.0316728 (PMC11781662; doi:10.1371/journal.pone.0316728)
Supplement: S5 Table — The table contains relative importance (∑), regression coefficient (b), Unconditional standard error (SE) and 95% confidence interval (CI) for b. Statistically significant predictors are in bold. (DOCX) [file pone.0316728.s005.docx]

**S5 Table: Model averaged parameter estimates for the top models for behavioral outcomes**. The table contains relative importance (∑), regression coefficient (b), Unconditional standard error (SE) and 95% confidence interval (CI) for b. Statistically significant predictors are in bold.

| **Outcomes** | **Predictors/levels** | **∑** | ***b*** | **SE** | **95% CI for *b*** |
| --- | --- | --- | --- | --- | --- |
| Proportion of moving scans | Phase_Pre-deworming | 1.00 | 0.04 | 0.16 | -0.28 to 0.37 |
|  | Phase_Early reinfection |  | -0.21 | 0.19 | -0.58 to 0.17 |
|  | **Phase_Late reinfection** |  | **-0.34** | **0.13** | **-0.58 to -0.08** |
|  | MPSR | 0.79 | -0.10 | 0.07 | -0.24 to 0.04 |
|  | Mean fGC | 0.34 | 0.02 | 0.04 | -0.06 to 0.10 |
|  | Sex_Male | 0.32 | 0.02 | 0.09 | -0.15 to 0.20 |
|  | Sex_AFI |  | -0.07 | 0.12 | -0.31 to 0.17 |
| Proportion of feeding scans | Phase_Pre-deworming | 0.63 | 0.09 | 0.10 | -0.11 to 0.28 |
|  | Phase_Early reinfection |  | -0.01 | 0.09 | -0.21 to 0.18 |
|  | Phase_Late reinfection |  | -0.09 | 0.11 | -0.31 to 0.11 |
|  | MPSR | 0.30 | 0.01 | 0.02 | -0.05 to 0.06 |
|  | Mean fGC | 0.64 | -0.06 | 0.06 | -0.17 to 0.06 |
|  | Sex_Male | 1.00 | -0.15 | 0.08 | -0.31 to 0.02 |
|  | **Sex_AFI** |  | **-0.38** | **0.091** | **-0.56 to -0.18** |
| Proportion of grooming scans | Phase_Pre-deworming | 1.00 | -0.02 | 0.20 | -0.42 to 0.38 |
|  | Phase_Early reinfection |  | 0.28 | 0.23 | -0.17 to 0.73 |
|  | **Phase_Late reinfection** |  | **1.39** | **0.15** | **1.11 to 1.69** |
|  | MPSR | 0.40 | 0.03 | 0.05 | -0.08 to 0.15 |
|  | Mean fGC | 0.25 | 0.01 | 0.03 | -0.05 to 0.06 |
|  | **Sex_Male** | 1.00 | **-2.44** | **0.34** | **-3.13 to -1.75** |
|  | Sex_AFI |  | -0.08 | 0.13 | -0.35 to 0.18 |
| Proportion of resting scans | Phase_Pre-deworming | 1.00 | -0.08 | 0.14 | -0.35 to 0.19 |
|  | Phase_Early reinfection |  | 0.17 | 0.16 | -0.14 to 0.48 |
|  | **Phase_Late reinfection** |  | **-0.71** | **0.13** | **-0.96 to -0.45** |
|  | MPSR | 0.37 | 0.03 | 0.05 | -0.07 to 0.12 |
|  | Mean fGC | 0.36 | -0.03 | 0.05 | -0.13 to 0.08 |
|  | Sex_Male | 0.17 | 0.03 | 0.09 | -0.15 to 0.21 |
|  | Sex_AFI |  | 0.01 | 0.06 | -0.11 to 0.14 |
