# Supplementary material for: Effects of gastrointestinal parasites on fecal glucocorticoids and behaviour in vervet monkeys (Chlorocebus pygerythrus)
Source: PLoS One. 2025 Jan 30;20(1):e0316728. doi: 10.1371/journal.pone.0316728 (PMC11781662; doi:10.1371/journal.pone.0316728)
Supplement: S6 Table — Predictors include month (June – December), and sex. The outcome variables include proportion of moving, feeding, grooming, and resting scans. (DOCX) [file pone.0316728.s006.docx]

**S6 Table. Results of ANOVA following generalized linear models (GLMs) for the behavioural outcome variables across months during the non-experimental year (2015) in vervet monkeys (Chlorocebus pygerythrus) at Lake Nabugabo, Uganda.** Predictors include month (June – December), and sex. The outcome variables include proportion of moving, feeding, grooming, and resting scans.

| **Outcomes** | **Predictors** | **Chisq** | **df** | **p-value** |
| --- | --- | --- | --- | --- |
| Proportion of moving scans | Month | 7.28 | 3 | 0.06 |
|  | Sex | 3.57 | 2 | 0.18 |
| Proportion of feeding scans | Month | 6.95 | 3 | 0.07 |
|  | Sex | 1.09 | 2 | 0.58 |
| Proportion of grooming scans | Month | 3.46 | 3 | 0.34 |
|  | Sex | 188.36 | 2 | <0.05* |
| Proportion of resting scans | Month | 0.97 | 3 | 0.81 |
|  | Sex | 2.59 | 2 | 0.27 |
