# Supplementary figures and images for: Effects of gastrointestinal parasites on fecal glucocorticoids and behaviour in vervet monkeys (Chlorocebus pygerythrus)
Source: PLoS One. 2025 Jan 30;20(1):e0316728. doi: 10.1371/journal.pone.0316728 (PMC11781662; doi:10.1371/journal.pone.0316728)

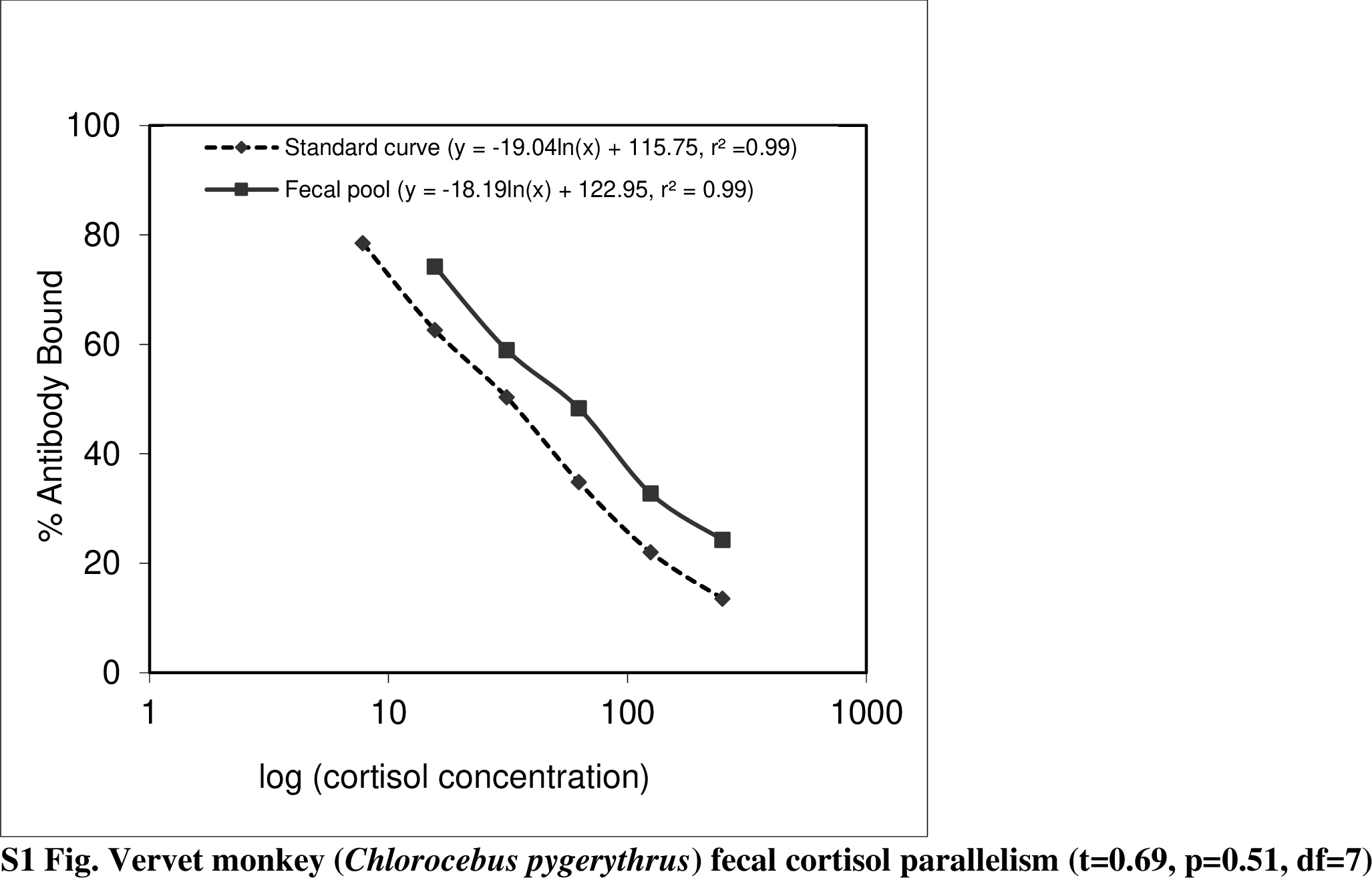

Supplement: S1 Fig — (TIF) [file pone.0316728.s007.tif]

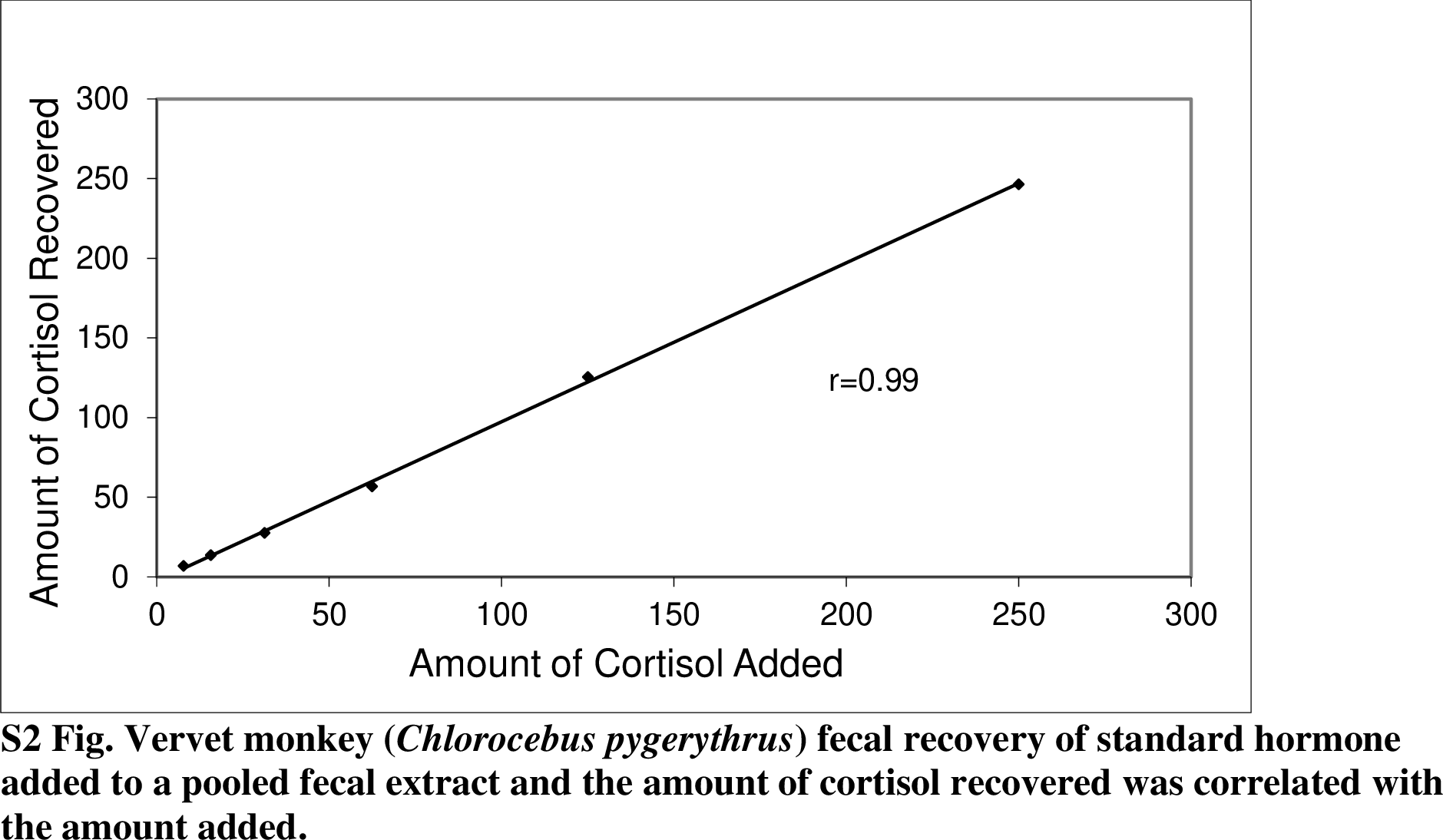

Supplement: S2 Fig — (TIF) [file pone.0316728.s008.tif]

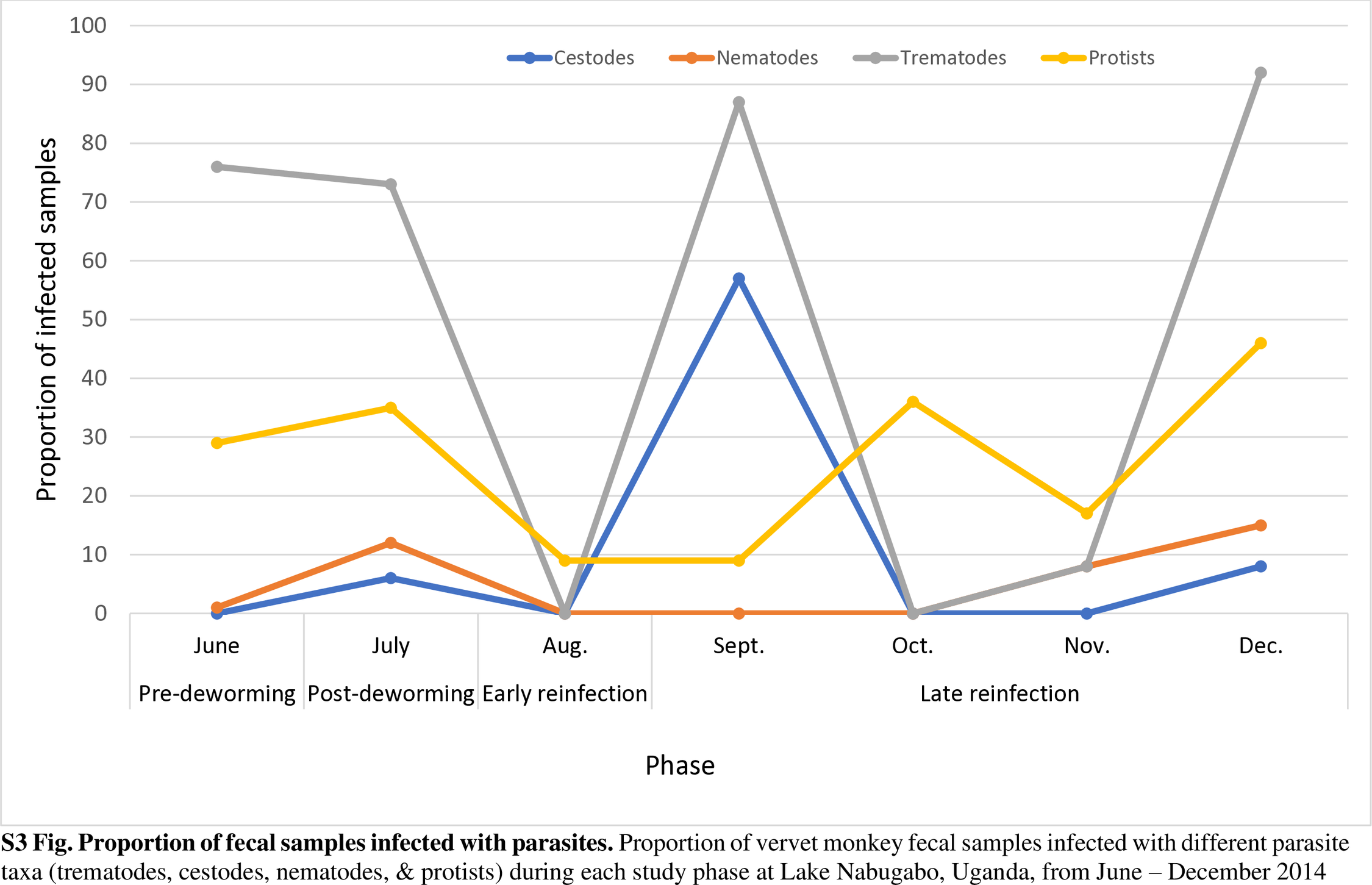

Supplement: S3 Fig — Proportion of infected samples with different parasite taxa (trematodes, cestodes, nematodes, & protists) during each study phase at Lake Nabugabo, Uganda, from June – December 2014. (TIF) [file pone.0316728.s009.tif]
