# Supplementary material for: Effects of gastrointestinal parasites on fecal glucocorticoids and behaviour in vervet monkeys (Chlorocebus pygerythrus)
Source: PLoS One. 2025 Jan 30;20(1):e0316728. doi: 10.1371/journal.pone.0316728 (PMC11781662; doi:10.1371/journal.pone.0316728)
Supplement: S1 Ethogram — (DOCX) [file pone.0316728.s010.docx]

**NABUGABO VERVET ETHOGRAM FOR ACTIVITY DATA**

**Breast feed (BF)** – actor suckles from one or both nipples simultaneously (Struhsaker 1967a); this is a behaviour directed by an infant (sometimes a young juvenile) to a female recipient; a.k.a., nursing or suckling.

**Chase (CH)** – Actor moves rapidly toward the recipient, who runs away (*sensu* “chase-and-attack” in Struhsaker 1967a); recipient frequently looks back at actor while running away, and often emits submissive vocalizations

- note: some chases may be “false” (sensu “false-chase” in Struhsaker 1967a), wherein the actor gallops slowly and hesitatingly towards the recipient that is never caught 🡪 actor generally subordinate to the recipient

**Copulate** – actor holds mountee hips with its hands, and legs with its feet (i.e., foot clasping); mount with thrusting; may be associated with grooming and individuals may alternate between role of actor and recipient; (note: mount may be incomplete, with grasping of only one body part Struhsaker 1967a)

**Drink** – individual is ingesting water from a ground or tree source (natural or man-made), normally involves lowering head towards standing water from water holes or rain pools; individuals may also lick water from hands, fingers, tree branches, or pelage of others (Struhsaker 1967a).

**Feed** – individual is manipulating and/or ingesting food.

- When possible, the observer should note the food species and part in the “comments”
- Food parts include: Ripe Fruit (RF), Unripe Fruit (UF), Unknown Fruit (FR), Flower (FL), Young Leaf (YL), Mature Leaf (ML), Leaf Bud (LB), Young Leaf Petiole (YLP), Mature Leaf Petiole (MLP), Bark, Dead Wood, Pine Needles, Seeds, Seed pods, Pith, Soil, Tuber/Root/Potato

**Groom (GR)** – actor combs through the fur of recipient using fingers and/or mouth, and may place foreign particles in mouth; may be accompanied by lipsmacking or teeth chattering (Struhsaker 1967a).

**Groom solicit (GS)** – actor presents a specific body part to recipient for grooming (Struhsaker 1967a)

**Mating presentation (PR)** – female actor stands quadrupedally and orients her hindquarters towards the male recipient, sometimes glancing over shoulder; female may stop to present if followed by a male or male grabs hindquarters of a sitting female (i.e., behaviour of “estrous female” as described in Struhsaker 1967a).

- Note: a male focal animal can therefore Receive a Present (RPR) from a female Interactant.

**Mating refusal (MR)** –female actor who is being grabbed by male remains sitting, lies down, crouches low, or walks away; note that female may also hit, lunge, or make “anti-copulatory squeal-scream” vocalizations (i.e., behaviour of “anestrous female” as described in Struhsaker 1967a).

**Move** – walking or galloping, occasionally including a hop, and may be preceded or interrupted by vigilance (Struhsaker, 1967a); locational displacement greater than 1 meter and excludes positional changes/adjustments with locational displacement of less than 1 meter (Schoof).

- Move between – moving between trees
- Move within – moving within a tree
- Move on ground – moving on the ground

**Out of sight (OO)** – focal animal is out of sight of the observer, usually resulting from substantial visual obstruction such as a building or dense vegetation, or because the focal animal has been “lost”.

**Play (PL; D, R, or M behaviour; or S for rare self-play)** – Play behaviours are quite variable and encompass a large number of behaviours described elsewhere (e.g., grab, chase, wrestle, mount, groom, embrace, hop, etc…)

- in play, these behaviours – such as chase or groom – are “alternated with one another in rapid sequence” and “may be the major distinctions between play and non-play encounters, rather than uniquely different behavior patterns” (Struhsaker 1967a: 33)
- intergroup play between juveniles and juvenile males of different groups may occur (Struhsaker 1967a).

**Rest** – individual is sitting in a relaxed (i.e., non-vigilant) manner; may also be lying on side or straddling a tree branch (Struhsaker 1967a)

**Scan** – individual is looking around, turning its head, in the farther visual distance >2meters; often occurs as a standing pause during a “move” (included visual foraging only during summer 2016 project); differs from “rest” because the animal is usually not sitting or lying down.

**Self-Groom** - an individual combs through its own pelage using fingers and/or mouth, and may place foreign particles in mouth (Schoof 2016, included scratching)

**Supplant (SU)** –the actor moves towards the recipient and occupies the space, eats the food, takes copulatory position, or assumes the grooming position of the recipient; the recipient or “supplantee” generally moves away, sometimes engaging in submissive behaviours such as lip-smacking or submissive vocalizations (Struhsaker, 1967a); a.k.a. displacement.

- Note: indicate in “comments” what the actor removed: food, grooming, or space (note that because “space” is generally taken when removing food or grooming from the recipient, “space” should only be used if neither food nor grooming were removed from the supplantee)

**Avoid (AV)** – at the approach of another individual (i.e., the recipient of the avoidance behaviour), the actor spontaneously vacates and moves away without any threat or aggression from the other individual; the recipient may just be passing by and does not occupy the space vacated by the actor (i.e., the individual doing the “avoiding”)

- note: if the space (or food/grooming partner) is then occupied by the approaching individual, this behaviour should be coded as a supplant directed by the approaching individual.

**VOCALIZATION (G or R; occasionally M)** – if you can identify the vocalization you may use the code below, otherwise use **VOC**

- **Alarm call (AC)** – Alarm calls are often multi-syllabic barks can be emitted in response to a real or perceived threat, most notably for “other vervet group”, snakes, eagles (i.e., *rraup*), leopards (i.e., *chirp*), & dogs and humans at Nabugabo (<http://www.psych.upenn.edu/~seyfarth/Baboon%20research/vervet%20vox.htm>)
- **Bark (BK)** – low-pitched and gruff uni-syllabic exhaled vocalization emitted by males during intergroup encounters and occasionally during intragroup agonism; given towards other vervet monkeys who are fighting, it is emitted to stop the fighting (Struhsaker, 1967b).
- **Chutter (CT)** – low-pitched, monotonal and staccato vocalization emitted by females and juveniles to express aggression and solicit assistance; mouth is closed and the teeth are covered (Struhsaker, 1967b).
- **Submissive vocalizations (SV)** – lipsmacking, teeth-chattering, purring (Struhsaker 1967b, Henzi 1982), and also includes:
  - ***Woof woof (WW)****:* This call is non-tonal, deep, and has a guttural sound (Struhsaker, 1967) emitted with closed or slightly-open mouth to indicate submission (Struhsaker, 1967b).
  - ***Wa (WA)****:* This call is a continuous tonal exhalation that occurs with a grimace and indicates submission (Struhsaker, 1967b); may be combined with as “Woof-wa” vocalization.
  - ***Rraugh (RR)****:* For this call the mouth is closed or partially opened and the teeth are covered (Struhsaker, 1967b). This call is emitted by yearlings when they approach older members of the group, and is a signal of nonaggression (Struhsaker, 1967b); includes both the long and short rraugh, and the aarr-rraugh.
  - ***Lipsmack (LS):*** Moving the lips together quickly, opening and closing the mouth repeatedly.
- **Squeal-Scream (SS):** high-pitched, piercing calls usually are emitted by females and juveniles that are seeking help from threats by an aggressor, and may be anti-copulatory (Struhsaker, 1967b).

**UNIDENTIFIED INTERACTANT(S)**

AM# – Adult male

AF# – Adult female without infant

AD# – Adult, unsexed

SM# – Subadult male

SF# – Subadult female

SB# – Subadult, unsexed

JM# – Juvenile Male

JF# – Juvenile Female

JV# – Juvenile, unsexed

IN# – infant (always unsexed)

XX# – Unidentified vervet

ZZ – other species (if known, indicate the species in the “comments”; e.g., snake, bird, dog, cow)

OBS – observer

HUM – other human

- If there are multiple interactants, use a comma to separate their IDs

ZRT- Red tailed monkey

BWC – Black and white colobus monkey

BBN - Baboon

DOG – Dog (indicate how many in the comments, and their behaviour)

COW – Cow

PIG – Pig

GOT – Goat

CKN – Chicken
